# Improving the Nonvolatile Memory Characteristics of Sol–Gel-Processed Y_2_O_3_ RRAM Devices Using Mono-Ethanolamine Additives

**DOI:** 10.3390/ma17215252

**Published:** 2024-10-28

**Authors:** Seongwon Heo, Soohyun Choi, Sangwoo Lee, Yoonjin Cho, Jin-Hyuk Bae, In-Man Kang, Kwangeun Kim, Won-Yong Lee, Jaewon Jang

**Affiliations:** 1 School of Electronic and Electrical Engineering, Kyungpook National University, Daegu 41566, Republic of Korea; pos03034@knu.ac.kr (S.H.); ctg999@naver.com (S.C.); sangw98@knu.ac.kr (S.L.); chongo815wls@knu.ac.kr (Y.C.); jhbae@ee.knu.ac.kr (J.-H.B.); imkang@ee.knu.ac.kr (I.-M.K.); 2 School of Electronics and Information Engineering, Korea Aerospace University, Goyang 10540, Republic of Korea; kke@kau.ac.kr; 3 The Institute of Electronic Technology, Kyungpook National University, Daegu 41566, Republic of Korea

**Keywords:** sol–gel, RRAM, mono-ethanolamine, oxygen vacancy, Y_2_O_3_, endurance, retention

## Abstract

In this study, Y_2_O_3_-based resistive random-access memory (RRAM) devices with a mono-ethanolamine (MEA) stabilizer fabricated using the sol–gel process on indium tin oxide/glass substrates were investigated. The effects of MEA content on the structural, optical, chemical, and electrical characteristics were determined. As the MEA content increased, film thickness and crystallite size decreased. In particular, the increase in MEA content slightly decreased the oxygen vacancy concentration. The decreased film thickness decreased the physical distance for conductive filament formation, generating a strong electric field. However, owing to the lowest oxygen vacancy concentration, a large electrical field is required. To ensure data reliability, the endurance cycles across several devices were measured and presented statistically. Additionally, endurance performance improved with the increase in MEA content. Reduced oxygen vacancy concentration can successfully suppress the excess formation of the Ag conductive filament. This simplifies the transition from the high- to the low-resistance state and vice versa, thereby improving the endurance cycles of the RRAM devices.

## 1. Introduction

Recently, resistive random-access memory (RRAM) devices have been considered the main component of next-generation memory systems. Firstly, the flash memory mainstream cannot last owing to its physical scaling limit [1]. Secondly, to handle mass information, neuromorphic devices that mimic the data processing mechanism of the human brain have been developed. Resistivity, which is a key electrical characteristic, of RRAM devices determines signal intensity and duration and can be controlled [2,3,4,5]. In addition, the structure of an RRAM device is simple, only consisting of three layers, i.e., the bottom electrodes, main active channel layer, and top electrodes. Extreme scalability with high performance and low power consumption can be expected from these devices [3,4,5].

For preparing active channel layers, several metal oxides, such as ZrO_2_, TiO_2_, Hf_X_O, and Y_2_O_3_, have been investigated [6,7,8,9,10,11]. These metal oxides are thermally and chemically stable with respect to organic channel layers. Moreover, these metal oxides do not encounter the alignment issue and are compatible with the complementary metal oxide semiconductor process, unlike nanowires, carbon nanotubes, graphene, or two-dimensional materials. Each of these metal oxides has advantages, and among them, Y_2_O_3_ exhibits promising properties. Y_2_O_3_ is an insulator with high k-values and a large optical bandgap. Therefore, Y_2_O_3_ may replace the low-k SiO_2_ insulator used in the semiconductor fabrication industry [12,13]. Recently, based on these advantages, GaN- and SiC-based power semiconductor devices have incorporated Y_2_O_3_ [14,15,16]. In addition, the high thermal and chemical stabilities and fast ion movement of Y_2_O_3_ render this metal oxide suitable for use in active channel layers in RRAM devices [17,18,19]. Finally, the presence of a sneak path leakage current arises as a problem when attempting to realize RRAM arrays with unit RRAM devices. To solve this problem, a transistor is connected to the RRAM devices, forming a 1T-1R structure [20,21]. Post-deposited Y_2_O_3_ layers present on metal-oxide thin-film transistors (TFTs) boost the bias stability of TFTs. When Y_2_O_3_ layers are simultaneously used as active channel layers of RRAMs and passivation layers of metal-oxide TFTs, the fabrication time and cost of the IT-IR structure can be drastically reduced [22,23].

Unlike the conventional vacuum-based deposition method, solution-based methods can be used to easily and rapidly deposit metal oxides without requiring time-consuming and high-cost vacuum pumps. Additionally, solution-based methods can be used to deposit metal-oxide layers for large-area applications. The starting materials used are liquid-phase precursors, indicating that the prepared liquid-phase solutions can be used for other next-generation mass-product fabrication processes such as roll-to-roll or in an inkjet printing system [24,25,26]. Sol–gel is a representative solution-based deposition method, which is simple and can be used to easily deposit high-quality metal oxides. In addition, by changing the precursors, stabilizers, and deposition or annealing temperature, the structural, chemical, and electrical characteristics of the obtained metal oxides can be easily modified. These characteristics considerably affect the electrical properties of metal-oxide-based electrical devices.

In this study, Y_2_O_3_-based RRAM devices were fabricated, and the sol–gel process was used for preparing Y_2_O_3_ layers. Additionally, the well-known capping material, mono-ethanolamine (MEA), was used, and the ratio between the amounts of precursors and MEA was varied. For the first time, the effects of the amount of MEA on the structural, chemical, optical, and electrical characteristics of these Y_2_O_3_ RRAM devices were investigated. In addition, a method to obtain uniform and oxygen-vacancy-less Y_2_O_3_ films using MEA was introduced. The fabricated Y_2_O_3_ RRAM devices exhibited conventional bipolar RRAM characteristics. In addition, the initial high-forming voltage process was unnecessary for forming the initial conductive filament. The Y_2_O_3_ films with a 20% molar ratio of MEA to the added precursor exhibited a cubic structure and the lowest oxygen vacancy concentration. The fabricated Y_2_O_3_ RRAM devices with this molar ratio exhibited a SET voltage of +0.64 V and a RESET voltage of −7.84 V, and the high- and low-resistance state ratio was over 10^3^. This device exhibited stable memory properties even after 10^3^ write and erase processes, and after changing states, the resistance states lasted for 10^4^ s without any significant deterioration.

## 2. Materials and Methods

Commercial indium tin oxide (ITO)-deposited glass substrates (Sigma-Aldrich, St. Louis, MO, USA) were used. The ITO/glass was sonicated in acetone for 10 min, followed by sonication in deionized water (DI) for an additional 10 min. To clean and activate the surfaces for improving adhesion, wettability, and overall process quality, these substrates were treated with ultraviolet/ozone for 1 h. A total volume of 10 mL of the Y_2_O_3_ precursor with a 0.3 M concentration was prepared by dissolving 1.04 g of Y(NO_3_)_3_·4H_2_O (Sigma-Aldrich, St. Louis, MO, USA, 99.9%) in 2-methoxyethanol (Sigma-Aldrich, St. Louis, MO, USA, 99.9%) as a solvent. Similarly, a separate 10 mL solution with a 0.3 M concentration of MEA (Sigma-Aldrich, St. Louis, MO, USA) was prepared by dissolving MEA in 2-methoxyethanol. Both solutions were sonicated for 10 min to ensure complete dissolution without any precipitation. The MEA-to-Y(NO_3_)_3_·4H_2_O molar ratio (x) was varied between 0–20. The prepared solutions were subsequently mixed in specific volume ratios to produce four different YMEA-x precursors, where x represents MEA molar ratios of 0%, 10%, and 20%. Subsequently, the solutions were stirred for 1 h at 80 °C to ensure homogeneous mixing and obtain clear solutions. The prepared precursors were subsequently spin-coated onto the cleaned ITO substrates by employing the sol–gel method under conditions of 3000 rpm for 50 s. After coating, the substrates were baked on a hot plate at 150 °C for 10 min, followed by annealing at 500 °C in a furnace for 2 h in air. Subsequently, Ag was deposited on the coated film to form top electrodes with a thickness of 100 nm using a thermal evaporator. The deposition was conducted under 5.0 × 10 ⁻⁶ Torr pressure with a 2.0 Å/s deposition rate, yielding 30 µm × 30 µm Ag electrodes. Field-emission scanning electron microscopy (FESEM, S-4800, Hitachi, Tokyo, Japan) was employed to assess the surface morphologies and thicknesses of the films. The crystal structures, orientations, and sizes of the films were analyzed using grazing-incidence X-ray diffraction (GIXRD, X’Pert Pro, Malvern PANalytical, Malvern, UK) with an incidence angle of 0.3° and Cu Kα radiations (λ = 0.154 nm). X-ray photoelectron spectroscopy (XPS; NEXSA, Thermo Fisher, Waltham, MA, USA) with monochromatic Al Kα radiation (1486.6 eV) was used to determine the elemental compositions and chemical states of the films. The surface roughness is obtained using scanning probe microscopy (SPM; Park NX20, Park Systems, Suwon, Republic of Korea; tapping mode). To evaluate the electrical characteristics of the fabricated RRAM devices, a probe station (MST T-40000A, Hwaseong, Republic of Korea) equipped with a source measurement unit (Keithley 2636B, Cleveland, OH, USA) was used.

## 3. Results and Discussion

**Figure 1:**
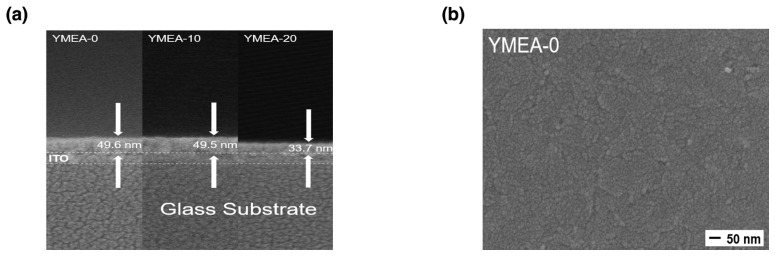

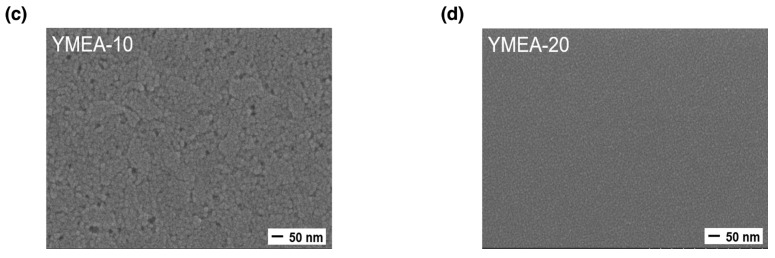
(**a**) Cross-sectional SEM image and (**b**–**d**) surface SEM images as a function of MEA content.

**Figure 2:**
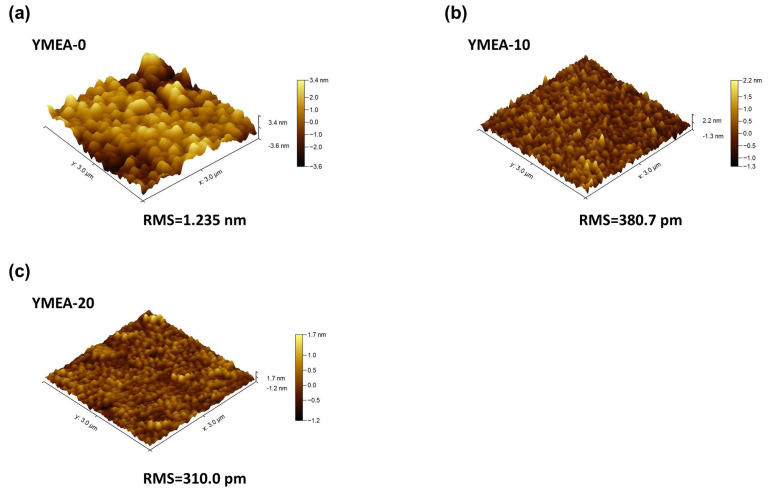
Three-dimensional SPM surface images of the Y_2_O_3_ films, as a function of MEA content: (**a**) YMEA-0, (**b**) YMEA-10, and (**c**) YMEA-20, respectively.

Figure 1a–d exhibit the FESEM images of the deposited films. The measured thicknesses of the YMEA-0 (Y_2_O_3_ only), -10, and -20 films were 49.6, 49.5, and 33.7 nm, respectively. The thicknesses of the films decreased up to YMEA-10. In addition, the YMEA-0 and YMEA-10 films contained wrinkles on their surfaces. However, these wrinkles disappeared and the surfaces became smooth (YMEA-20). The wrinkles originated from the relaxation of stress in the deposited films. Owing to the different thermal expansions between Y_2_O_3_ and ITO and the decrease in the capillary force of the liquid phase, compressive stress was generated, thereby forming wrinkles on these surfaces [27]. As compressive stress decreased, film thickness decreased; therefore, YMEA-20 contained a flat and smooth surface. In addition, the added MEA stabilized particle growth and suppressed particle agglomeration, yielding even distributions and generating even and uniform surfaces [28]. The SPM surface images of the films are presented in Figure 2 and the measured root-mean-square surface roughness (R_p_) for films was 1.235 nm, 380.7 pm, and 310.0 pm, respectively. The smoothness of the film surfaces increases as the MEA ratio increases.

**Figure 3:**
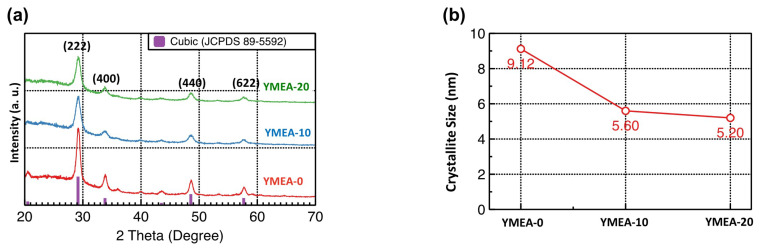
(**a**) GIXRD data and (**b**) calculated crystallite sizes of the Y_2_O_3_ films as a function of MEA content.

GIXRD analysis, the results of which are presented in Figure 3, is employed to determine whether the crystalline structure, crystallite size, and tensile and compressive stresses of the films changed owing to MEA mixing. The YMEA-0 film exhibited a (222)-oriented polycrystalline phase. The diffraction peaks observed at 2θ values of 20.45°, 29.15°, 33.78°, 35.90°, 39.84°, 43.48°, 48.53°, and 57.61° correspond to the (211), (222), (400), (411), (332), (134), (440), and (622) planes, respectively, of the Y_2_O_3_ cubic structure (JCPDS 88-2162). Monoclinic metastable Y_2_O_3_ can be formed at relatively low temperatures [29]. However, all the Y_2_O_3_ films formed herein exhibited cubic structures. The maximum peak observed at 29.30° suggested that the grains primarily developed in the (222) plane. In contrast, the GIXRD patterns of the YMEA-10 and -20 films displayed slightly broad diffraction peaks. The crystallite sizes, which can be calculated from these patterns, were determined using the Scherrer equation.

(1) $$D=\frac{0.9\;\lambda }{\beta \;Cos\;\theta },$$

where D represents the crystallite size, λ represents the wavelength, i.e., 0.154 nm, of the Cu Kα radiation, β is the full width at half maximum of the main peak, and θ is the peak position. The calculated crystallite sizes corresponding to the (222) plane were 9.12 (YMEA-0), 5.60 (YMEA-10), and 5.20 nm (YMEA-20). Nucleation and grain growth are the main mechanisms involved in the crystallization process. When the film thickness is small, the surface energy suppresses the grain growth [30]. Therefore, under the same 500 °C annealing conditions, YMEA-20 was more difficult to crystallize than YMEA-0 and YMEA-10. In addition, the main peaks did not shift as a function of the MEA content, indicating that the interlayer distance (d) remained the same, regardless of changes in the MEA content.

**Figure 4:**
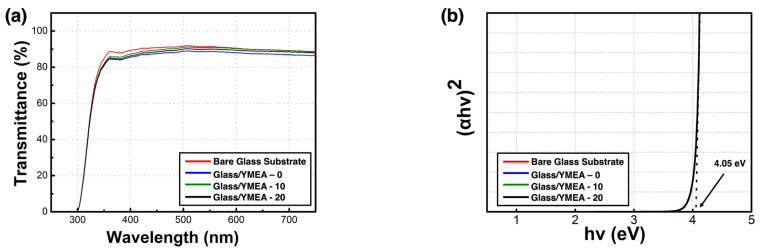
(**a**) Transmittance spectra of the glass/ITO/Y_2_O_3_ films and (**b**) (αhν)^2^ versus hν plots of the Y_2_O_3_ films as a function of MEA content.

The optical characteristics of the glass/Y_2_O_3_ films are displayed in Figure 4. The transmittance spectra of the Y_2_O_3_ films and bare glass substrates, encompassing the visible-light spectrum in the range of 380–780 nm, are displayed in Figure 4a. The glass/Y_2_O_3_ films demonstrated an average transmittance exceeding 80% in the visible-light range, comparable to that of the glass substrate. The films exhibited similar transparency regardless of the MEA concentration. The optical bandgaps of the Y_2_O_3_ films were estimated from the slopes of the linear regions in the (αhν)^2^ versus hν graphs. Figure 4b displays these plots derived from the Tauc equation.

(αℎ*v*)^*n*^ = *A*(ℎ*v*−*E*_*g*_), (2)

where α represents the absorption coefficient, ℎ is Planck’s constant, *ν* refers to the frequency, and E_g_ denotes the optical bandgap. The films exhibited an optical bandgap of approximately 4.05 eV, consistent across different fabrication conditions.

**Figure 5:**
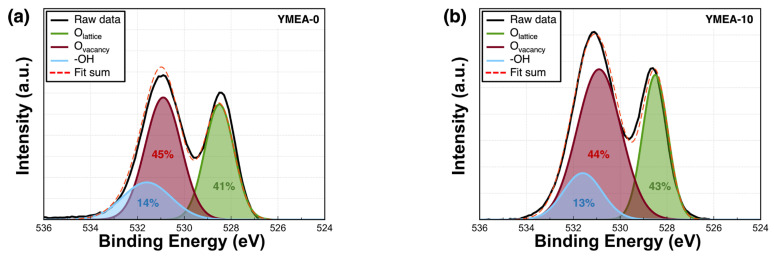

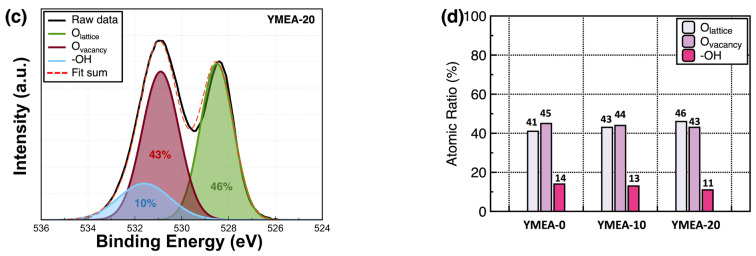
(**a**–**c**) XPS O1s spectra of the Y_2_O_3_ films and (**d**) chemical composition variations as a function of MEA content.

Figure 5 depicts the chemical compositions of the films obtained via XPS. The XPS peaks were fitted using a Gaussian distribution. This analysis method was employed to accurately determine the chemical compositions of the films. Charges were corrected using the C1s peak at 285.0 eV. Figure 5a–d show the O1s core-level spectra. The O1s peak consists of three components corresponding to the oxygen lattice (O_L_), oxygen vacancies (O_V_), and hydroxyl groups (–OH), with binding energies of 528.5, 530.9, and 531.6 eV, respectively. The proportion of hydroxyl groups decreased from 13.6% (YMEA-0) to 13.5% (YMEA-10) and then to 10.7% (YMEA-20). Furthermore, the films with added stabilizers exhibited lower O_V_ contents than the reference, decreasing from 44.5% (YMEA-0) to 44.4% (YMEA-10) and subsequently to 43.5% (YMEA-20). In contrast, the oxygen lattice component increased from 41.0% (YMEA-0) to 42.0% (YMEA-10) and subsequently to 46.0% (YMEA-20). The proportions of the three O peaks in the O 1 s spectra of the Y_2_O_3_ films as a function of MEA content are listed in Table 1. The metal–oxygen bond weakened and the number of point defects related to the oxygen vacancies increased. A certain amount of added MEA can substitute O with N in the films, decreasing the oxygen vacancy concentration [31].

**Figure 6:**
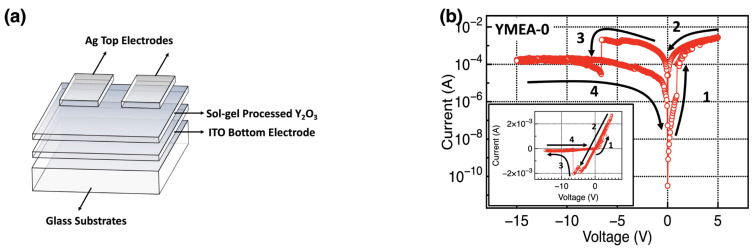

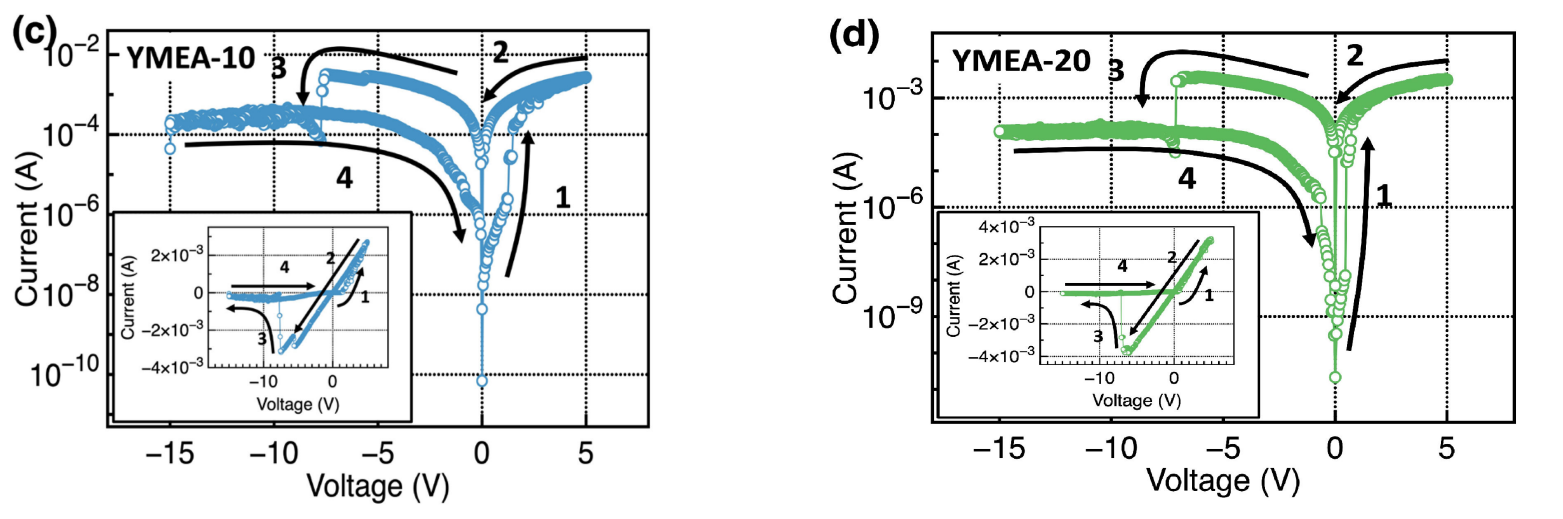
(**a**) Schematic image of the fabricated Y_2_O_3_ RRAM devices. (**b**–**d**) Representative I–V curves of the Y_2_O_3_ RRAM devices as a function of MEA content. The arrows and numbers indicate the voltage sweep directions.

Figure 6 displays the schematic and electrical characteristics of the fabricated Y_2_O_3_ RRAM device. The current–voltage (I–V) curves of several devices tested under identical conditions are superimposed, highlighting the typical bipolar resistive switching behavior of the devices. The fabricated Y_2_O_3_ RRAM devices did not require the initial forming process implemented by applying large voltage biases via the top electrodes. Oxygen-vacancy-rich oxides or Ag or Cu top electrodes do not require the initial forming process [32,33]. These devices can switch between high- and low-resistance states (HRS and LRS, respectively) when subjected to suitable voltage pulses, facilitating data storage. Initially, the RRAM devices began in the HRS. When a positive voltage bias was applied to the top Ag electrodes, oxidation occurred and Ag^+^ ions were generated (Ag → Ag^+^ + e^−^). These ions moved to the bottom electrodes owing to the electrical field. At the interface between Y_2_O_3_ and the bottom ITO electrodes, the drifted Ag^+^ ions received electrons and started to form a conductive Ag filament. When the formed Ag filament touched the top Ag electrodes, the resistance state changed to the LRS. The SET voltage is the required positive voltage to switch from the HRS to the LRS. Inversely, when a negative voltage bias was applied to the top Ag electrodes, the formed Ag conductive filaments were broken, changing the resistance state to the HRS. The RESET voltage is the required negative voltage to switch from the LRS to the HRS.

**Figure 7:**
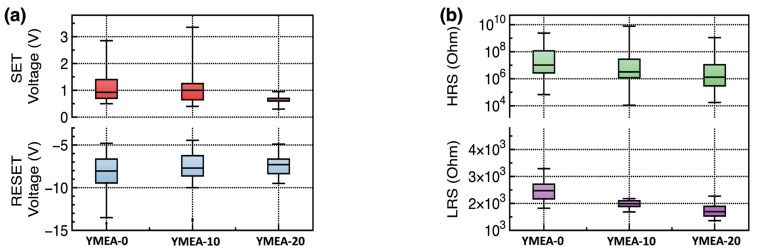
(**a**) Obtained performance parameters of the RRAM devices: (**a**) SET and RESET voltages and (**b**) HRS and LRS.

The performance parameters of the RRAM devices are extracted and plotted, as shown in Figure 7. The SET voltage values of YMEA-20 were slightly lower and more uniform than those of the other devices. The electric field required for the SET process can be calculated using the following equation:

(3) $$E_{eff}=\frac{\Delta \phi }{q\times t_{ox}}+\frac{V_{SET}}{t_{ox}}$$

where ∆Φ is the work function difference between the two electrodes, q is the amount of charge, V_SET_ is the SET voltage of the fabricated RRAM device, and t_ox_ is the Y_2_O_3_ film thickness. The work functions of the top Ag and bottom ITO electrodes were 4.26 and 4.70 eV, respectively. Under these values, the work function difference between the two electrodes was 0.44 eV. The measured film thicknesses are 49.6, 49.5, and 33.7 nm, as determined from the cross-sectional SEM images shown in Figure 1. The calculated effect fields of the RRAM devices were 0.24, 0.23, and 0.32 MV·cm^−1^. Even though the film thickness of YMEA-20 was the lowest, the YMEA-20 RRAM required the highest SET voltages. This indicates that to form the conductive Ag filament, a higher electric field was required than those in the other cases. The oxygen vacancies within the Y_2_O_3_ films assisted in Ag^+^ migration. As the oxygen vacancy concentration increased, the ease of Ag^+^ migration and formation of the conductive filament increased. In addition, if the film was rough, locally induced electrical fields simplified the formation of conductive filaments [34,35,36]. Therefore, the YMEA-20 devices, having the lowest oxygen vacancy concentration and a smooth morphology, required the highest electric field to form a conductive filament.

**Figure 8:**
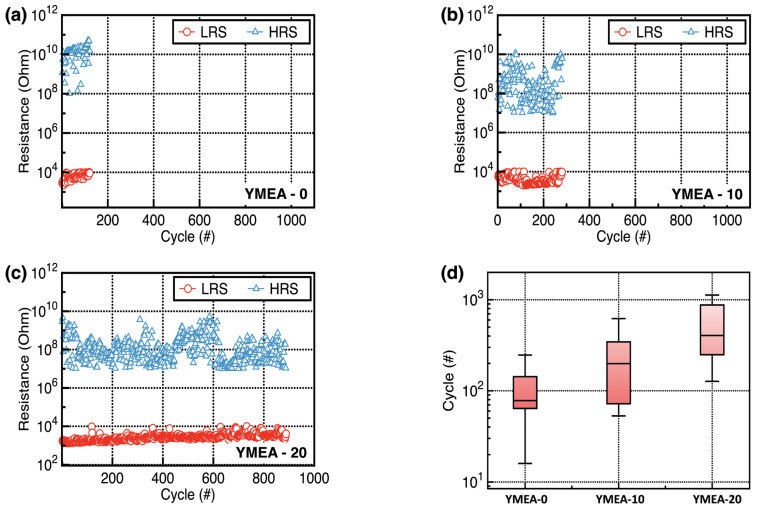
(**a**–**c**) Representative endurance data, as a function of MEA content and (**d**) statistical data of the obtained endurance cycles.

**Figure 9:**
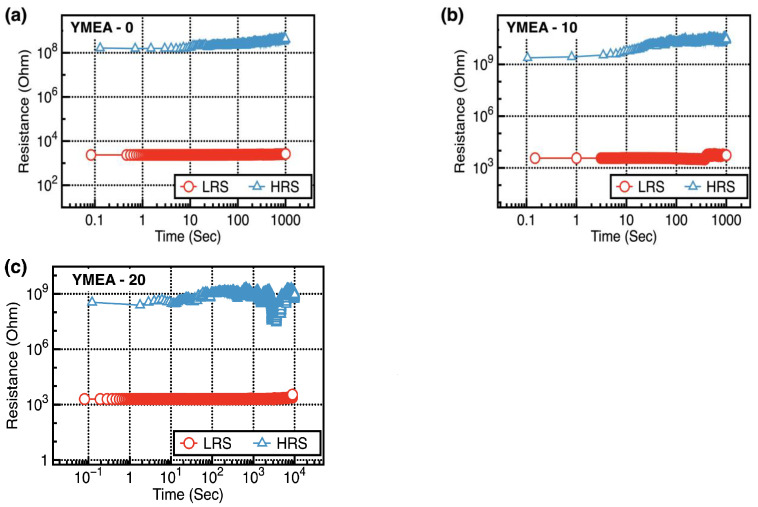
Representative retention data as a function of MEA content: (**a**) YMEA-0, (**b**) YMEA-10 and (**c**) YMEA-20, respectively.

The nonvolatile memory characteristics of the fabricated RRAM devices were estimated by obtaining endurance and retention data. Figure 8a–c display the representative endurance characteristics of the fabricated Y_2_O_3_ RRAM devices. Figure 8d displays statistical data showing the maximum endurance cycles as a function of MEA content. During endurance tests, the LRS and HRS were measured at V_read_ = +0.1 V after programming (+5.0 V for 500 ms) and erasing (−15.0 V for 500 ms). The RRAM devices consisting of the Y_2_O_3_ films (YMEA-20) demonstrated superior endurance properties. In addition, the YMEA-20 devices exhibit stable retention properties up to 10^4^ s without significant changes in the LRS and HRS values, as shown in Figure 9. Excess formation of the conductive filament made triggering the SET and RESET operations increasingly challenging. Relatively high oxygen vacancy concentrations in the YMEA-0 and YMEA-10 films formed excess conductive filaments, which complicated the change of status during the endurance tests.

## 4. Conclusions

In this study, Y_2_O_3_-based RRAM devices containing an MEA stabilizer were fabricated using the sol–gel process on ITO/glass substrates and investigated. For the first time, the effects of the MEA content on the structural, chemical, optical, and electrical characteristics of these Y_2_O_3_ RRAM devices were determined. In addition, a method to form uniform and oxygen-vacancy-less Y_2_O_3_ films using MEA was introduced. Optimizing the MEA content decreased the oxygen vacancy concentration and film thickness. This reduced oxygen vacancy concentration can successfully suppress the excess formation of the Ag conductive filament. Firstly, this reduced the SET and RESET voltages, improving uniformity. Secondly, the fabricated Y_2_O_3_-based RRAM devices, with optimized MEA content, exhibited improved endurance characteristics, a critical parameter deciding the nonvolatile memory properties. This simplified the transition from HRS to LRS and vice versa, thereby improving the endurance cycles and retentions of the RRAM devices. These results elucidate the effects of the capping material and the oxygen vacancy concentration on RRAM device performances.

## Figures and Tables

**Table 1:** Oxygen compositions of the Y_2_O_3_ films as a function of MEA content.

	Oxygen State	O_L_	O_V_	–OH
	Binding Energy (eV)	528.5	530.9	531.6
YMEA-0	Area (%)	41.0	45.0	14.0
YMEA-10	Area (%)	43.0	44.0	13.0
YMEA-20	Area (%)	46.0	43.0	11.0

## Data Availability

Data are available in a publicly accessible repository.
